# Polymer Fiber Rigid Network with High Glass Transition Temperature Reinforces Stability of Organic Photovoltaics

**DOI:** 10.1007/s40820-024-01442-0

**Published:** 2024-06-18

**Authors:** Qiao Zhou, Cenqi Yan, Hongxiang Li, Zhendong Zhu, Yujie Gao, Jie Xiong, Hua Tang, Can Zhu, Hailin Yu, Sandra P. Gonzalez Lopez, Jiayu Wang, Meng Qin, Jianshu Li, Longbo Luo, Xiangyang Liu, Jiaqiang Qin, Shirong Lu, Lei Meng, Frédéric Laquai, Yongfang Li, Pei Cheng

**Affiliations:** 1 College of Polymer Science and Engineering, State Key Laboratory of Polymer Materials Engineering, Sichuan University, Chengdu, 610065 People’s Republic of China; 2 KAUST Solar Center, Physical Science and Engineering Division, King Abdullah University of Science and Technology (KAUST), Thuwal, Kingdom of Saudi Arabia; 3 Beijing National Laboratory for Molecular Sciences, CAS Key Laboratory of Organic Solids, Institute of Chemistry, Chinese Academy of Sciences, Beijing, 100190 People’s Republic of China; 4 Department of Material Science and Technology, Taizhou University, Taizhou, 318000 People’s Republic of China

**Keywords:** Inverted organic photovoltaics, Thermal stability, Aramid nanofibers, Morphology control, Charge carrier dynamics

## Abstract

Organic photovoltaics (OPVs) need to overcome limitations such as insufficient thermal stability to be commercialized. The reported approaches to improve stability either rely on the development of new materials or on tailoring the donor/acceptor morphology, however, exhibiting limited applicability. Therefore, it is timely to develop an easy method to enhance thermal stability without having to develop new donor/acceptor materials or donor–acceptor compatibilizers, or by introducing another third component. Herein, a unique approach is presented, based on constructing a polymer fiber rigid network with a high glass transition temperature (*T*_g_) to impede the movement of acceptor and donor molecules, to immobilize the active layer morphology, and thereby to improve thermal stability. A high-*T*_g_ one-dimensional aramid nanofiber (ANF) is utilized for network construction. Inverted OPVs with ANF network yield superior thermal stability compared to the ANF-free counterpart. The ANF network-incorporated active layer demonstrates significantly more stable morphology than the ANF-free counterpart, thereby leaving fundamental processes such as charge separation, transport, and collection, determining the device efficiency, largely unaltered. This strategy is also successfully applied to other photovoltaic systems. The strategy of incorporating a polymer fiber rigid network with high *T*_g_ offers a distinct perspective addressing the challenge of thermal instability with simplicity and universality.

**Supplementary Information:**

The online version contains supplementary material available at 10.1007/s40820-024-01442-0.

## Highlights

- A unique approach is proposed: constructing a polymer fiber rigid network with high glass transition temperature.
- Frozen bulk heterojunction morphology impeded deterioration of exciton quenching, charge transport, and charge extraction properties during thermal aging.
-  The strategy is universal and can be further optimized for enhanced thermal stability and improved mechanical resilience.

**Supplementary Information:**

The online version contains supplementary material available at 10.1007/s40820-024-01442-0.

## Graphical abstract

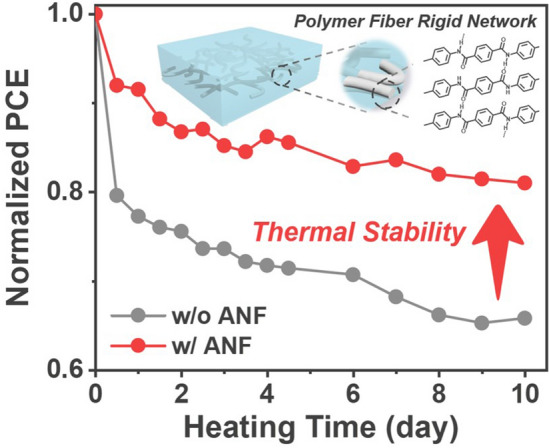

## Introduction

Organic photovoltaics (OPVs) have garnered considerable attention due to their unique advantages such as lightweight, flexibility, semitransparency, cost-effectiveness, and relative ease of mass production. These attributes position them as promising contenders in the realm of clean and renewable energy [1–11].

Despite notable progress in improving efficiency [12–27], the challenge of thermal stability hampers the commercialization of OPVs as a technology [28–33]. The thermal stability of active layers is of importance; it encompasses the thermal stability of the material itself and the stability of the bulk heterojunction (BHJ) morphology. The large *π*-conjugated structure of organic photovoltaic materials grants them a much higher thermal decomposition temperature than the operating temperature. However, the BHJ active layer morphology remains instable and susceptible to heat. Donor and acceptor molecules are inclined to diffuse upon heating, resulting in the alteration of molecular orientation, crystallization, and phase separation. These changes ultimately impact the efficiency-determining processes, including exciton dissociation, charge transport, and charge collection within devices [34–36].

In the last decade, researchers have made huge efforts to enhance the thermal stability of OPVs [37–53]. The design and synthesis of photoactive materials with lower molecular diffusivity to enhance stability is one such approach. Another widely used approach is the addition of a donor–acceptor compatibilizer to stabilize the donor–acceptor interface and prevent phase-separated domains from coalescing. Additionally, thermal stability can be improved by adding a discrete third component, either to stabilize or to modify the morphology. However, the aforementioned methods often involve the design and synthesis of new materials or require the donor:acceptor (D:A) system to have a wide processing window, with limited applicability in wide-scale production. Therefore, it is timely and important to develop a simple method to enhance the thermal stability without having to develop and synthesize a new donor/acceptor pair or donor–acceptor compatibilizer, or by introducing another, ill-controlled third component.

Herein, we propose a unique methodology using incorporation of a polymer fiber rigid network with high glass transition temperature (*T*_g_) to immobilize the active layer morphology, impede the movement of acceptor and donor molecules, and thereby improve device thermal stability. One-dimensional aramid nanofibers (ANF) derived from poly (*p*-phenylene terephthalamide) (PPTA) fibers [54, 55] not only retain their remarkable mechanical properties, notable thermostability, excellent electrical insulation capabilities, and high chemical resistance, but also exhibit nanoscale dimensions and a large aspect ratio, making them particularly suitable for incorporation into organic photoactive layers with a typical thickness of around 100 nm. We prepared and utilized one-dimensional ANF as the network material. The inverted OPV device with an ANF network yields a comparable power conversion efficiency (PCE) of 16.9% and retains as much as 94% of its efficiency after 3 h at 130 °C, while the device without an ANF network only preserves 82% after heating. In-depth morphology characterization confirms the stability of the sole ANF network and, notably, reveals significantly improved morphological stability in the active layer incorporating the ANF network, as compared to the active layer without the ANF network. This stable morphology reduces deterioration of charge separation, transport, and extraction properties, resulting in significantly enhanced thermal stability. The universality of this approach is demonstrated by the thermal stability improvement of other fullerene-based and non-fullerene-based photovoltaic systems after ANF network incorporation. The utilization of this solution-processable, interspace-tunable, rigid network represents a universal strategy to enhance the thermal stability of any OPVs.

## Experimental Section

### Materials

PM6, PTB7-Th, BTP-eC9, L8-BO, IEICO-4F, and PC_71_BM were purchased from Solarmer Materials Inc. ANF was synthesized according to the procedures in the literature [56]. Chlorobenzene and MoO_3_ were purchased from J&K Scientific, Inc. Silver was purchased from ZhongNuo Advanced Material (Beijing) Technology Co., Ltd. The other materials and solvents were common commercial level and used as received.

### Device Fabrication

The structure of all OPVs adopts the inverted device structure, namely ITO/ZnO/active layer/MoO_3_/Ag structure. The ITO glass substrates were sonicated sequentially in detergent, deionized water, acetone, and isopropanol for 30 min, respectively. After drying, the ITO substrates were UV/ozone-treated for 20 min. The ZnO precursor solution was prepared by dissolving 300 mg of zinc acetate dihydrate and 137.5 μL of ethanolamine in 5 mL of 2-methoxyethanol. After being stirred constantly for 12 h, the precursor solution was spin-coated at 3000 rpm onto the ITO surface, followed by annealing at 200 °C for 1 h. For ANF-introduced devices, the different concentrations of ANF solution were spin-coated onto the ZnO layer at 3000 rpm, followed by annealing at 200 °C for 1 h. The used active layer of PM6:BTP-eC9 (1:1.2, wt:wt) was dissolved in chlorobenzene with a total concentration of 22 mg mL^−1^ and stirred at 80 °C for 2 h in a nitrogen glovebox. Then the DIO additive was added to the solution with a volume ratio of 0.7%. The film thickness was controlled at about 100 nm. The fabricated active layer was followed with thermal annealing at 100 °C for 5 min.

Besides, the other three photovoltaic layers, consisting of three different photovoltaic systems (shown in Fig. 6 later), were dissolved in chlorobenzene (PTB7-Th:IEICO-4F and PM6:PC_71_BM) or chloroform (PM6:L8-BO) with various weight ratios and spin-coated on top of the ZnO or ANF layer. Finally, a layer of 2.5 nm MoO_3_ and then an Ag layer of 100 nm were evaporated subsequently under vacuum at 2 × 10^−4^ Pa. The device area was 0.1 cm^2^ (with a mask area of 0.0392 cm^2^).

### Instruments and Characterizations

Ultraviolet–visible–near infrared (UV–vis-NIR) absorption was measured with the SHIMADZU UV-2600I spectrophotometer. Scanning electron microscopy (SEM) studies analyses were performed using Apreo S HiVoc of Thermo Fisher Scientific (FEI). The *J*-*V* curves were measured under a computer-controlled Keysight B2900 source meter under 100 mW cm^−2^, AM 1.5 G solar simulator (Enlitech SS-X50). The *J*-*V* curves were measured with forward scan mode from −0.5 to 1.2 V, and the scan step was 0.02 V with a dwell time of 0.01 s. The light intensity was calibrated by a standard silicon solar cell to give a range from 0.99 to 1.01 sun. The light intensity dependence plot was obtained from the solar simulator’s built-in shutter with the intensity ranging from 2% to 100%. The EQE spectra were received from a commercial QE measurement system (Taiwan, Enlitech, QE-R).

#### GIWAXS and GISAXS Measurements

GIWAXS data were obtained at beamline BL02U2 of Shanghai Synchrotron Radiation Facility (SSRF). The monochromatic of the light source was 1.24 Å. The data were recorded by using the two-dimensional image plate detector of Pilatus 2 M from Dectris, Switzerland.

GISAXS data of films were obtained at beamline BL16B1 of Shanghai Synchrotron Radiation Facility (SSRF). The monochromatic of the light source was 1.24 Å. The incidence angle was 0.2°, and the sample-to-detector distance was 2200 mm by calibration for GISAXS. The GISAXS 1D profiles were fitted with a universal model following Eq. 1. Data fitting was done using SasView (version 5.01) software.

1 $${\text{I}}\left( {\text{q}} \right) = \frac{{{\text{A}}_{1} }}{{\left[ {1 + \left( {{\text{q}}\xi } \right)^{2} } \right]^{2} }} + {\text{A}}_{2} {\text{ P}}\left( {{\text{q}},{\text{R}}} \right){\text{ S}}\left( {{\text{q}},{\text{R}},\eta ,{\text{D}}} \right) + {\text{B}}$$

2 $${\text{S}}\left( {\text{q}} \right) = 1 + \frac{{{\text{sin}}\left[ {\left( {{\text{D}} - 1} \right){\text{tan}}^{ - 1} \left( {{\text{q}}\eta } \right)} \right]}}{{\left( {{\text{qR}}} \right)^{{\text{D}}} }}\frac{{{\text{D}}\Gamma \left( {{\text{D}} - 1} \right)}}{{\left[ {1 + \frac{1}{{\left( {{\text{q}}\eta } \right)^{2} }}} \right]^{{\left( {{\text{D}} - 1} \right)/2}} }}$$

where *A*_*1*_, *A*_*2*_, and *B* are independent fitting parameters and q is the scattering wave vector. The average correlation length *ξ* of the PM6 domain and the Debye–Anderson–Brumberger (DAB) term make up the first term I*(q)*. The contribution from BTP-eC9 fractal-like aggregations is seen in the second term S*(q)*. Here, *R* is the mean spherical radius of the primary BTP-eC9 particles, P(*q*, *R*) is the form factor of the BTP-eC9, S(*q*, *R*, *η*, *D*) is the fractal structure factor to explain the primary particles interaction in this fractal-like aggregation system, *η* is the correlation length of the fractal-like structure, and *D* is the fractal dimension of the network. Equation 3 was used to calculate the average domain size by the Guinier radius of the fractal-like network R_*g*_.

3 $${\text{R}}_{g} = \eta \sqrt {\frac{{{\text{D}}\left( {{\text{D}} + 1} \right)}}{2}}$$

#### TRPL Measurement

The TRPL samples were excited with the wavelength-tunable output of a Chameleon Ultra Laser (Coherent, central wavelength 820 nm) at 725 nm. The repetition rate of the fs pulses was 80 MHz, and typical pulse energies were in the range of 500 nJ. The PL of the samples was collected by an optical telescope (consisting of two plano-convex lenses) and focused on the slit of a spectrograph (PI Spectra Pro SP2300) and detected with a Streak Camera (Hamamatsu C10910) system with a temporal resolution of 1.4 ps. The data were acquired in photon counting mode using the Streak Camera software (HPDTA) and exported to Origin Pro 2020 for further analysis [57–59].

#### TPV and TPC Measurements

The transient photovoltage (TPV) measurement was conducted under 1 sun illumination with a white light-emitting diode, and the device was set to the open-circuit condition, while the device was set to the short-circuit condition in the dark for the transient photocurrent (TPC) measurement. The output signal was collected by keysight oscilloscope for both TPV and TPC [60, 61].

#### Photo-CELIV Measurement

In the Photo-CELIV measurement, the OPVs were fabricated with the same method as mentioned above. The data were obtained by the all-in-one characterization platform, Paios (Fluxim AG, Switzerland). The delay time is set to 0 s, the light intensity is 100%, the light-pulse length is 100 µs, and finally, the sweep ramp rate rises from 20 to 100 V ms^−1^ [62].

## Results and Discussion

### Material Properties

**Fig. 1:**
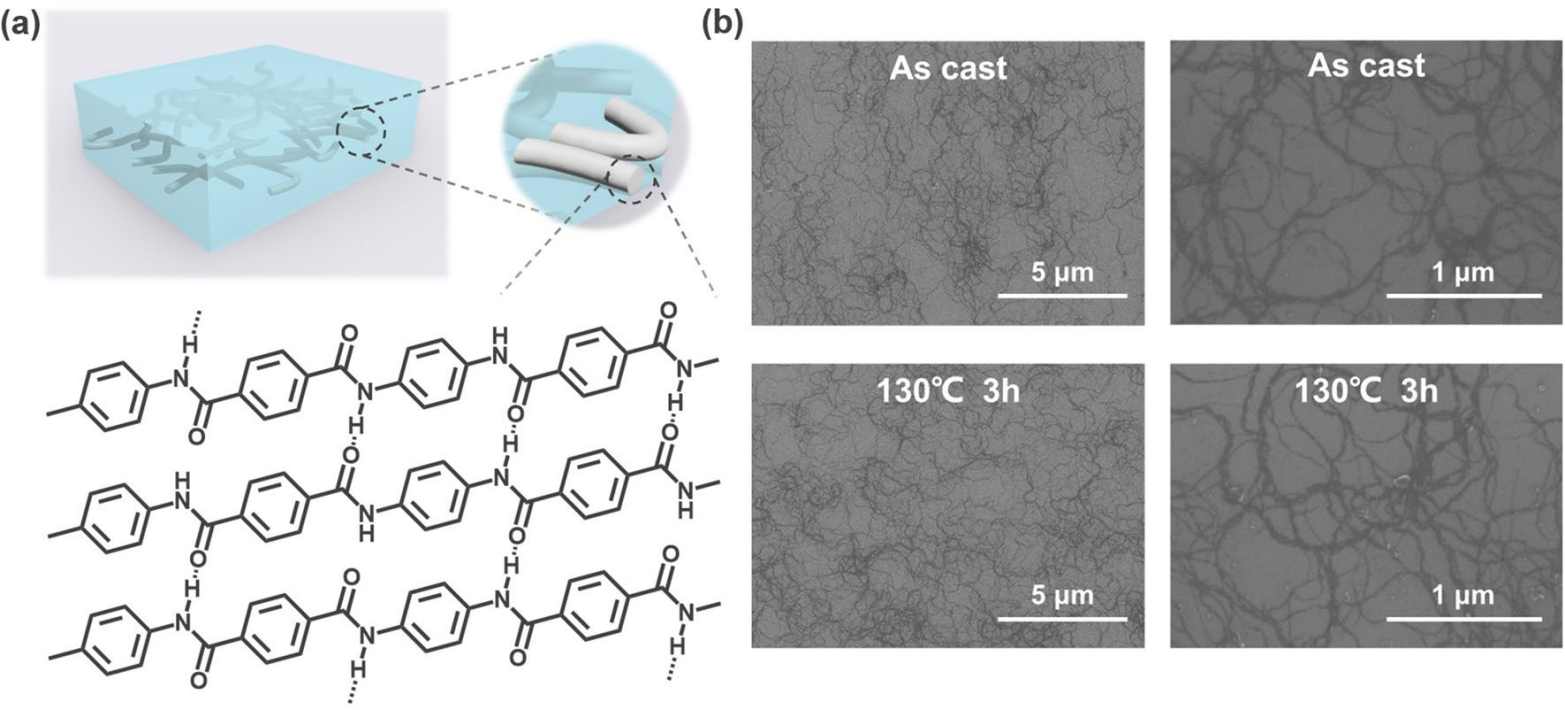
**a** Schematic illustration of ANF networks in active layers and molecular structure with hydrogen-bonding interaction of ANF. **b** SEM images of ANF films prepared from 0.46 mg mL^−1^ dispersion before and after heating at 130 °C for 3 h

By implementing a deprotonation technique, we disrupted the hydrogen bond network between the PPTA polymer chains, leading to the fragmentation of macroscopic PPTA fibers into individual nanofibers known as ANF [51]. These polymer fiber-derived ANFs possess extended and para-linked rigid molecular chains, resulting in high *T*_*g*_ surpassing 300 °C [63]. Upon spin coating of an ANF dispersion onto a substrate, a nanoscale interspaced rigid network is formed, as depicted in Fig. 1a. This network serves as a suitable network for the subsequent deposition of donor/acceptor blends. SEM images reveal that ANF exhibits nanoscale dimensions with a diameter ranging from 20 to 30 nm and possesses a large aspect ratio (Fig. 1b). Moreover, the ANF network exhibits minimal morphological changes after continuous heating at 130 °C. Figure S1 illustrates the distribution of ANF at different dispersion concentrations on the substrate, showing the formation of a denser ANF network as the concentration increases from 0.23 to 0.91 mg mL^−1^. As shown in Fig. S2, the ANF network shows very high transmittance across the wavelength range of 300–1100 nm. Therefore, the incorporation of the ANF network into the active layer of the device exerts negligible impact on light absorption, ensuring efficient utilization of incident light. As shown in the atomic force microscopy (AFM) images (Fig. S3), the root-mean-square roughness (*R*_q_) of the active layer with and without ANF are very close (2.08 *vs.* 1.90 nm), indicating that the incorporation of ANF negligibly affects the surface smoothness of the active layer.

### Photovoltaic Performance and Thermal Stability

**Fig. 2:**
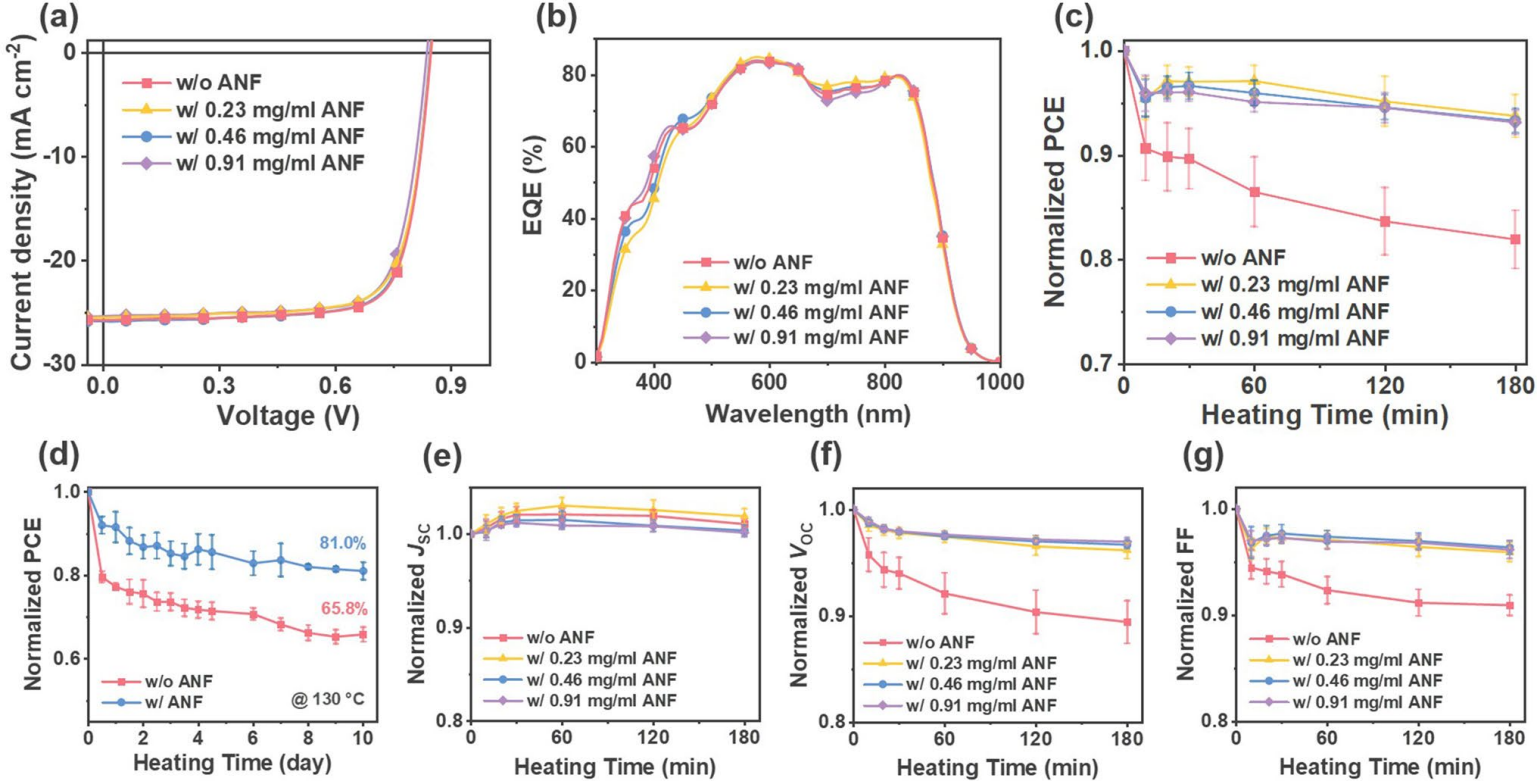
**a** The current density–voltage *J*-*V* characteristics, **b** external quantum efficiency EQE spectra, **c** (3 h-) **d** (10 days-) thermal stability, and **e–g** degradation of photovoltaic parameters for OPVs based on PM6:BTP-eC9 without and with ANF network at different dispersion concentrations. Degradation experiments were performed under a nitrogen atmosphere while subjecting the devices to heating at 130 °C

To assess the photovoltaic performance, inverted OPV devices were fabricated using the architecture: indium tin oxide (ITO)/zinc oxide (ZnO)/PM6:BTP-eC9/molybdenum trioxide (MoO_3_)/silver (Ag). The ANF network was deposited between ZnO and the photoactive layer through spin coating. Detailed information regarding device fabrication can be found in the Experimental Section. Figure 2a shows the current density–voltage (*J*-*V*) curves of the optimized devices without (w/o) or with (w/) the incorporation of the ANF network; relevant photovoltaic parameters are summarized in Table 1. The optimized devices without an ANF network exhibited a PCE of 17.0%, resulting from an open-circuit voltage (*V*_OC_) of 0.847 V, a short-circuit current density (*J*_SC_) of 26.2 mA cm^−2^, and a fill factor (FF) of 76.7%. The measured *J*_SC_ value was consistent with the *J*_SC_ value derived from the external quantum efficiency (EQE) spectra (Fig. 2b and Table 1). For the devices incorporating the ANF network, the PCEs were 16.5%, 16.9%, and 16.5% for devices with ANF concentrations of 0.23, 0.46, and 0.91 mg mL^−1^, respectively. The devices prepared with an ANF network concentration of 0.46 mg mL^−1^ exhibited moderate network formation and efficiency similar to those without ANF.

**Table 1:** Detailed photovoltaic parameters of PM6:BTP-eC9-based OPVs, including devices without ANF network and those with ANF network at various dispersion concentrations

ANF	*V*_OC_ (V)^a^	*J*_SC_ (mA cm^−2^)^a^	*J*_cal_ (mA cm^–2^)^b^	FF (%)^a^	PCE (%)^a^
None	0.847 (0.844 ± 0.002)	26.2 (26.2 ± 0.2)	25.2	76.7 (76.0 ± 0.4)	17.0 (16.8 ± 0.2)
0.23 mg/mL	0.846 (0.842 ± 0.003)	26.0 (25.9 ± 0.3)	25.1	75.1 (74.2 ± 0.7)	16.5 (16.2 ± 0.2)
0.46 mg/mL	0.845 (0.844 ± 0.002)	26.4 (26.1 ± 0.3)	25.2	75.9 (75.8 ± 0.3)	16.9 (16.7 ± 0.2)
0.91 mg/mL	0.837 (0.836 ± 0.004)	25.9 (26.1 ± 0.2)	25.1	76.1 (74.6 ± 0.9)	16.5 (16.3 ± 0.1)

^a^Average value of eight independent devices in parentheses

^b^Integrated from EQE value

The thermal stability of the devices was further investigated by performing stability tests at 130 °C under nitrogen atmosphere. The variation of the normalized photovoltaic parameters over the heating time is illustrated in Fig. 2c–g, with detailed data in Table S1. For devices without the ANF network, the average PCE gradually dropped to 86.5%, 83.7%, and finally 82.0% of the initial value after constant heating for 1, 2, and 3 h, respectively. In contrast, devices incorporating the ANF network exhibited an average efficiency loss of only 6.24%–6.86% after 3 h of heat exposure, indicating much improved thermal stability (Fig. 2c). Furthermore, as displayed in Fig. 2d, after 10 days (240 h) of thermal aging at 130 °C, the average PCE of devices without ANF decreased by 34.2%, while the ANF-incorporated devices exhibited an average efficiency loss of less than 20%. Analysis of the *J*_SC_ decay curves (Fig. 2e) revealed that during the thermal aging process, the *J*_SC_ value of all four devices exhibited a slight increase before stabilizing around the original level, without any discernible decline. The *V*_OC_ and FF parameters experienced reductions of 10.6% and 9.08%, respectively, in devices without the ANF network after keeping the devices at 130 °C for 3 h (Fig. 2f, g). Conversely, ANF-incorporated devices demonstrated less than 5% reduction in both *V*_OC_ and FF under the same conditions. A significant reduction in efficiency after heat treatment is observed in the inverted OPV without ANF, which is ascribed to a more pronounced reduction in *V*_OC_ and FF therein. These results indicate the crucial role of ANF in enhancing the heat resistance of OPVs.

### Film Morphology

**Fig. 3:**
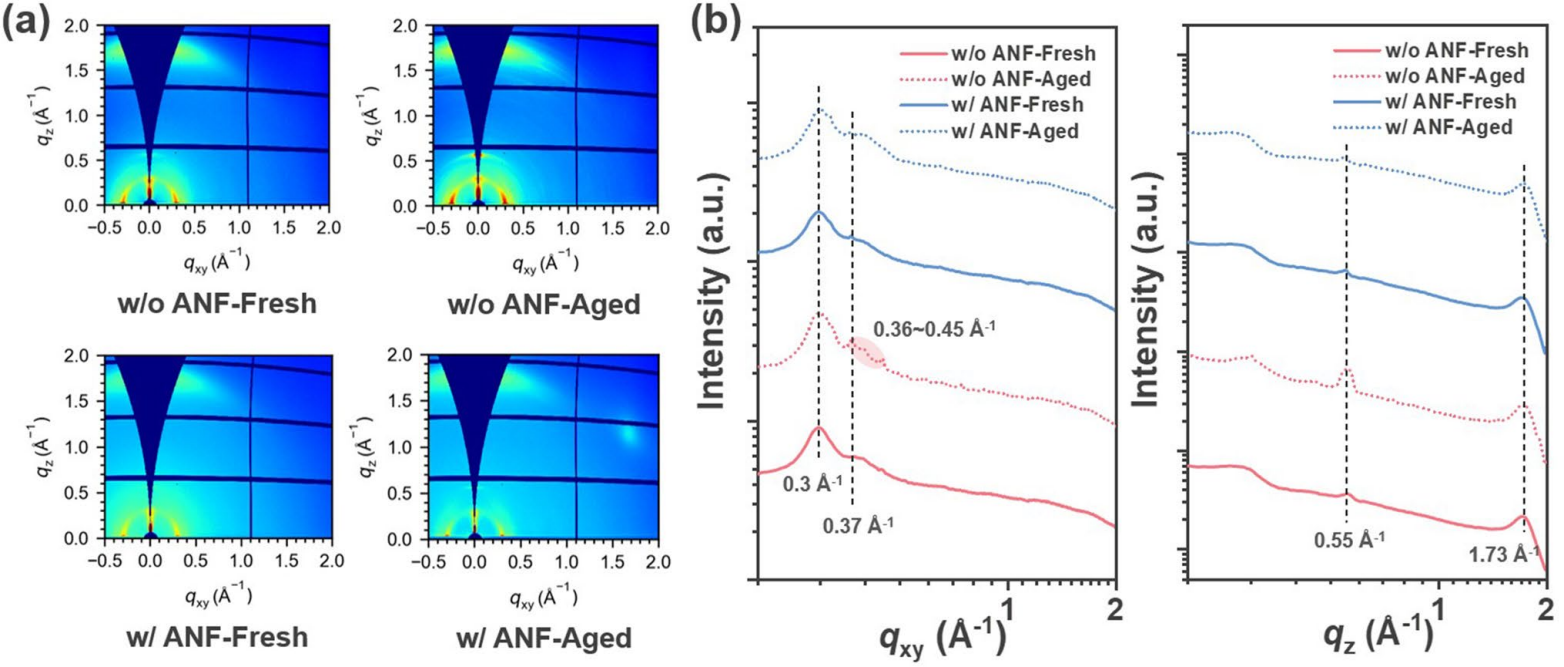
**a** GIWAXS 2D patterns and **b** 1D profiles of PM6:BTP-eC9 blend films without and with ANF network before and after heating at 130 °C for 3 h

To assess the morphological changes before and after continuous heating, grazing-incidence wide-angle X-ray scattering (GIWAXS) and grazing-incidence small-angle X-ray scattering (GISAXS) techniques were employed. Figure 3 presents the two-dimensional (2D) GIWAXS patterns and corresponding intensity profiles of PM6:BTP-eC9 blend films without and with ANF before and after heating at 130 °C for 3 h. The fresh samples without and with ANF exhibited similar characteristics, with *π–π* stacking (010) peaks at ~ 1.73 Å^−1^ in the out-of-plane (OOP) direction and lamellar stacking (100) peaks at ~ 0.3 Å^−1^ in the in-plane (IP) direction. Apart from that, the peaks at ~ 0.55 Å^−1^ in the OOP direction and ~ 0.37 Å^−1^ in the IP direction were attributed to the partially ordered microstructure of BTP-eC9 [64–66]. All of these peaks originated from PM6 and BTP-eC9, as confirmed by measurements on PM6 and BTP-eC9 neat films (Fig. S3). It illustrates the addition of ANF into the blend film had a negligible impact on the molecular orientation before heating. The neat ANF film displayed no diffraction peak in the 2D-GIWAXS pattern before and after heat treatment (Fig. S3), indicating its excellent heat resistance. The GIWAXS analysis revealed distinct differences between the ANF-free blend and the ANF-incorporated blend upon thermal aging. For the ANF-free blend, multiple sharp scattering peaks appeared along the IP direction at 0.36–0.45 Å^−1^ and the intensity of the peaks at *q*_xy_ = 0.3 Å^−1^ and *q*_z_ = 0.55 Å^−1^ increased, indicating the formation of a more ordered molecular structure and enhanced crystallinity after thermal treatment. In contrast, the ANF-incorporated blend maintained the original packing throughout the thermal aging process, as evidenced by the negligible change in GIWAXS diffraction peaks. These findings demonstrate that the rigid ANF network effectively impedes molecular diffusion within the photoactive layer of OPVs upon heating.

**Fig. 4:**
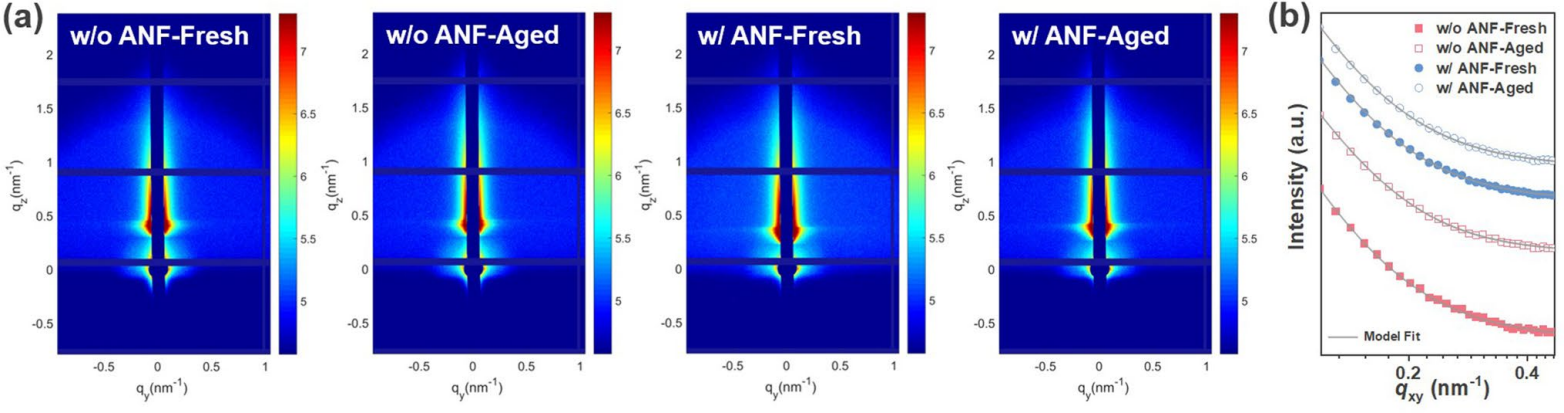
**a** GISAXS 2D patterns and **b** 1D profiles of PM6:BTP-eC9 blend films without and with ANF before and after heating at 130 °C for 3 h. The solid gray lines are the fitted curves using the Debye–Anderson–Brumberger (DAB) model and the fractal-like model

GISAXS was employed to investigate the evolution of domain sizes during thermal aging. The 2D patterns and the corresponding IP intensity profiles are presented in Fig. 4a, b. The profiles were fitted by the *Debye*–*Anderson*–*Brumberger* (DAB) model and the fractal-like model (Experimental Section), and the fitting parameters are summarized in Table 2. Here, the correlation length (*ξ*) represents the domain size of the PM6-rich phase, while *η* and *D* correspond to the correlation length and fractal dimension of the BTP-eC9 phase, respectively. The domain size of acceptor aggregation is denoted as 2*R*_g_. The fresh films, both without and with the ANF, exhibited a similar distribution of donor and acceptor domain sizes, resulting in comparable *ξ* and 2*R*_g_ value. This finding aligns with the observations from GIWAXS.

**Table 2:** Fitting parameters of 1D GISAXS profiles for corresponding blend films

Devices	*ξ* (nm)	*η* (nm)	*D*	2*R*_*g*_ (nm)
w/o ANF-fresh	22.1	6.7	2.9	31.9
w/o ANF-aged	33.7	7.3	3.2	37.8
w/ ANF-fresh	22.8	6.9	2.9	32.8
w/ ANF-aged	25.1	7.1	3.0	34.8

Subsequently, upon continuous heating, the ANF-free film displayed a substantial increase in the size of donor-rich and acceptor-rich domains. The *ξ* and 2*R*_g_ value increased from 22.1 to 33.7 nm and from 31.9 to 37.8 nm, respectively. The apparent increase in phase separation leads to a reduction of the donor/acceptor interface. In contrast, the domain size of the ANF-incorporated films increased only slightly, with *ξ* and 2*R*_g_ values increasing from 22.8 to 25.1 nm and from 32.8 to 34.8 nm, respectively.

**Scheme 1:**
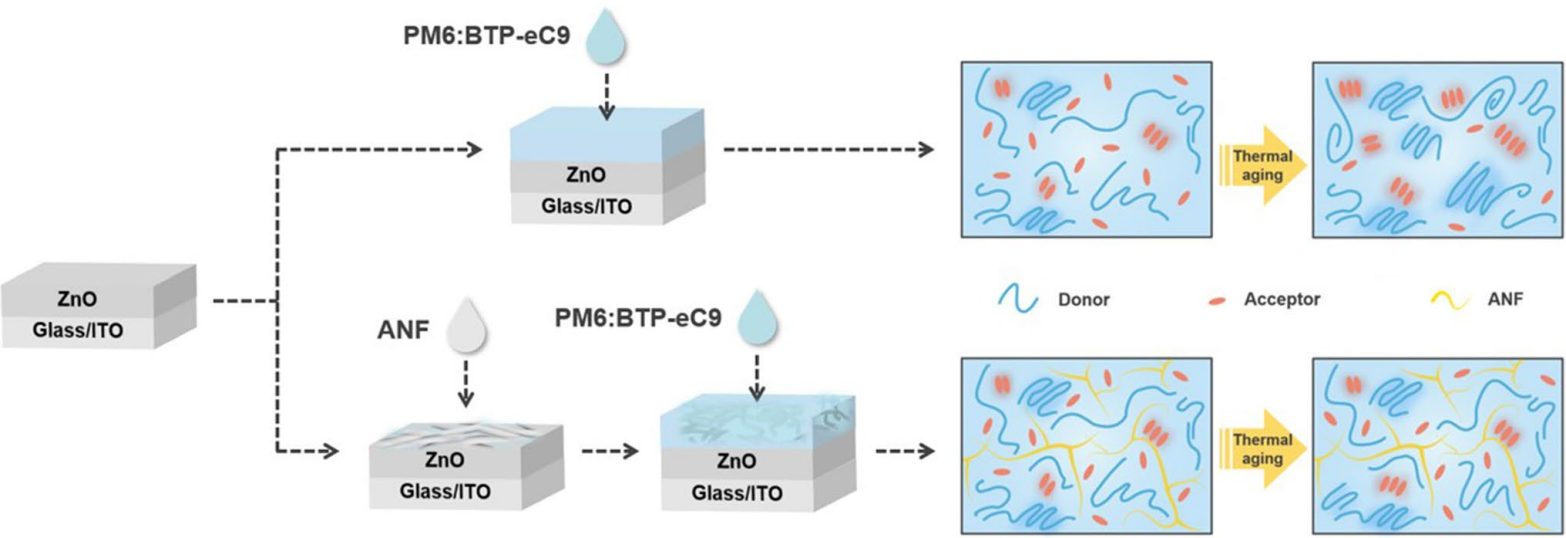
Schematic illustration of deposition process and morphological evolution of active layers without and with ANF network during thermal aging

The GIWAXS and GISAXS characterization provide direct evidence that the introduction of the ANF rigid network to the BHJ blend effectively impedes thermally induced molecule diffusion, and hinders further phase separation. This finding is consistent with the observed stable photovoltaic performance of ANF-incorporated devices. To visualize the function of the rigid ANF network in the active layer, the morphology evolution of films without and with the ANF network during thermal aging is illustrated by Scheme 1. In the absence of ANF, the donor and acceptor diffused upon heating, leading to significant aggregation. On the contrary, with the incorporation of ANF into the blend, molecule diffusion and aggregation are suppressed due to the spatial confinement imposed by the ANF rigid network. As a result, the ANF-incorporated active layer achieves higher morphological stability after prolonged heating compared to the ANF-free one.

### Exciton and Charge Carrier Dynamics

**Fig. 5:**
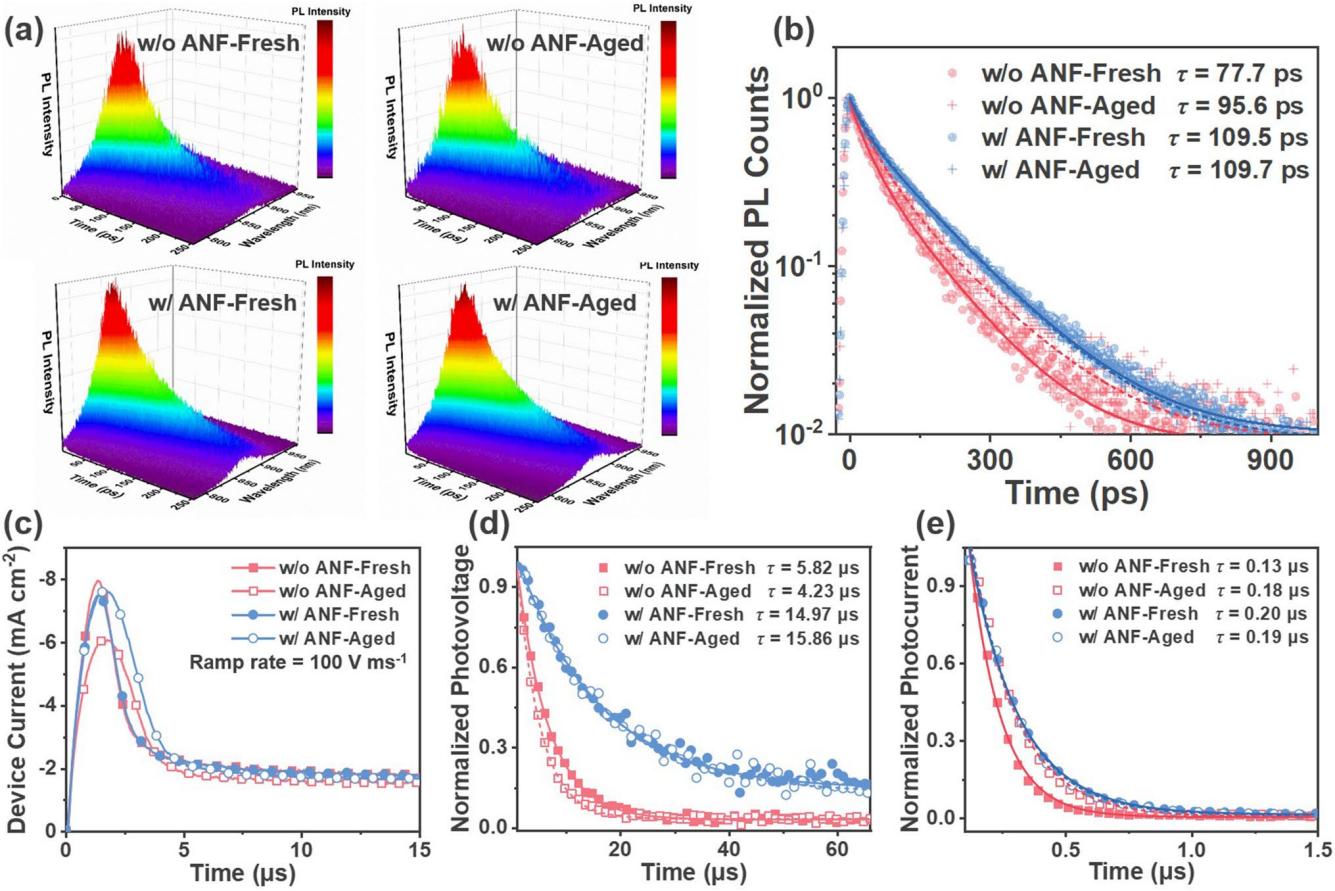
**a** 3D plots of the normalized TRPL data. **b** TRPL kinetics of PM6:BTP-eC9 blend films without and with ANF before and after heating at 130 °C for 3 h. **c** Photo-CELIV curves of the devices without and with ANF before and after heating at 130 °C for 3 h. **d** TPV and **e** TPC decay curves of corresponding devices

A series of photophysical measurements were carried out to study the photon-to-electron conversion processes and to relate those to photovoltaic properties and morphological features before and after thermal aging. Light harvesting was investigated through ultraviolet–visible (UV–Vis) absorption spectroscopy, as illustrated in Fig. S4. The UV–Vis absorption spectra of PM6:BTP-eC9 blend films without and with ANF were virtually similar. A slight red shift in the absorption peaks was observed after thermal aging. However, the spectra of all films remained virtually unaltered. The findings reveal a negligible difference in the light harvesting properties of all four films. Subsequently, the influence of ANF on the exciton dynamics was investigated via pico- to nanosecond time-resolved photoluminescence (TRPL) spectroscopy. The photoluminescence (PL) decay was characterized following pulsed photoexcitation at 725 nm. A bi-exponential decay function was used to parameterize the PL decay and to extract the amplitude-averaged decay times. The fitting details have been shown in our previous works [67–69]. The corresponding normalized TRPL data of all four films are presented as 3D plots in Fig. 5a with a clear PL emission peak from BTP-eC9. The maximum of the BTP-eC9 emission peak was tracked to further analyze the PL transients of the respective blend films. Upon heating to 130 °C for 3 h, the photoluminescence lifetime of the ANF-free blend film increased from 77.7 to 95.6 ps, indicating reduced exciton quenching. This can be a result of aggregation and crystallization and demixing in the aged ANF-free photoactive layer. In contrast, the ANF-incorporated blend film exhibited virtually the same photoluminescence lifetime (109.5 vs. 109.7 ps) before and after heating, suggesting effective preservation of exciton quenching, pointing to a thermally stable morphology of the ANF-incorporated blend.

The charge carrier mobilities in the device were evaluated by photoinduced charge carrier extraction using linearly increasing voltage (photo-CELIV) measurement. The measured mobilities for the as-cast devices, without and with ANF, are 3.70 × 10^−4^ and 3.43 × 10^−4^ cm^2^ V^−1^ s^−1^, respectively (Fig. 5c). The corresponding aged devices without and with ANF exhibited mobilities of 2.36 × 10^−4^ and 2.33 × 10^−4^ cm^2^ V^−1^ s^−1^, respectively. It is noteworthy that the degradation of the charge transport characteristics of the devices upon heating was mitigated by the incorporation of the ANF rigid network. These collective findings align with the more stable *V*_OC_ and FF in ANF-incorporated devices under elevated temperatures.

To gain a deeper understanding of charge extraction and recombination properties in the devices, transient photovoltage/photocurrent (TPV/TPC) measurements were conducted. The charge carrier lifetimes were determined from the TPV decay dynamics at open-circuit conditions, and a mono-exponential model was employed for fitting, as depicted in Fig. 5d. After 3 h of heating to 130 °C, the charge carrier lifetime (*τ*) of the aged device without ANF was shortened to 4.23 µs compared to the as-cast device (*τ* = 5.82 µs), suggesting an increase in charge recombination due to heating-induced morphological changes. Conversely, the as-cast and aged ANF-incorporated devices displayed comparable (or even slightly increased) charge carrier lifetimes (14.97 vs. 15.86 µs), consistent with the stable *V*_OC_ and FF. To investigate the competition between charge carrier sweep-out and recombination during device operation, TPC measurements were performed (Fig. 5e). The photocurrent decay time was 0.13 µs for the as-cast ANF-free device, while the aged ANF-free sample exhibited a longer decay time of 0.18 µs, indicating the presence of more traps in the ANF-free device aged at elevated temperatures. In contrast, thermal aging had a less detrimental effect on the charge extraction processes of ANF-incorporated devices, with decay times of 0.20 and 0.19 µs for as-cast and aged ANF-incorporated devices, respectively. Based on the aforementioned analysis, it can be concluded that ANF-processed devices exhibited largely unaltered charge carrier dynamics, which explains the reduced loss in *V*_OC_ and FF compared to ANF-free devices upon heating. Based on morphology and photophysics characterizations, the morphology achieved by the ANF network maintains efficient charge separation, transport, and extraction, thereby effectively safeguarding the photovoltaic performance against thermal degradation.

### Universality of the Rigid Network Introduced Strategy

**Fig. 6:**
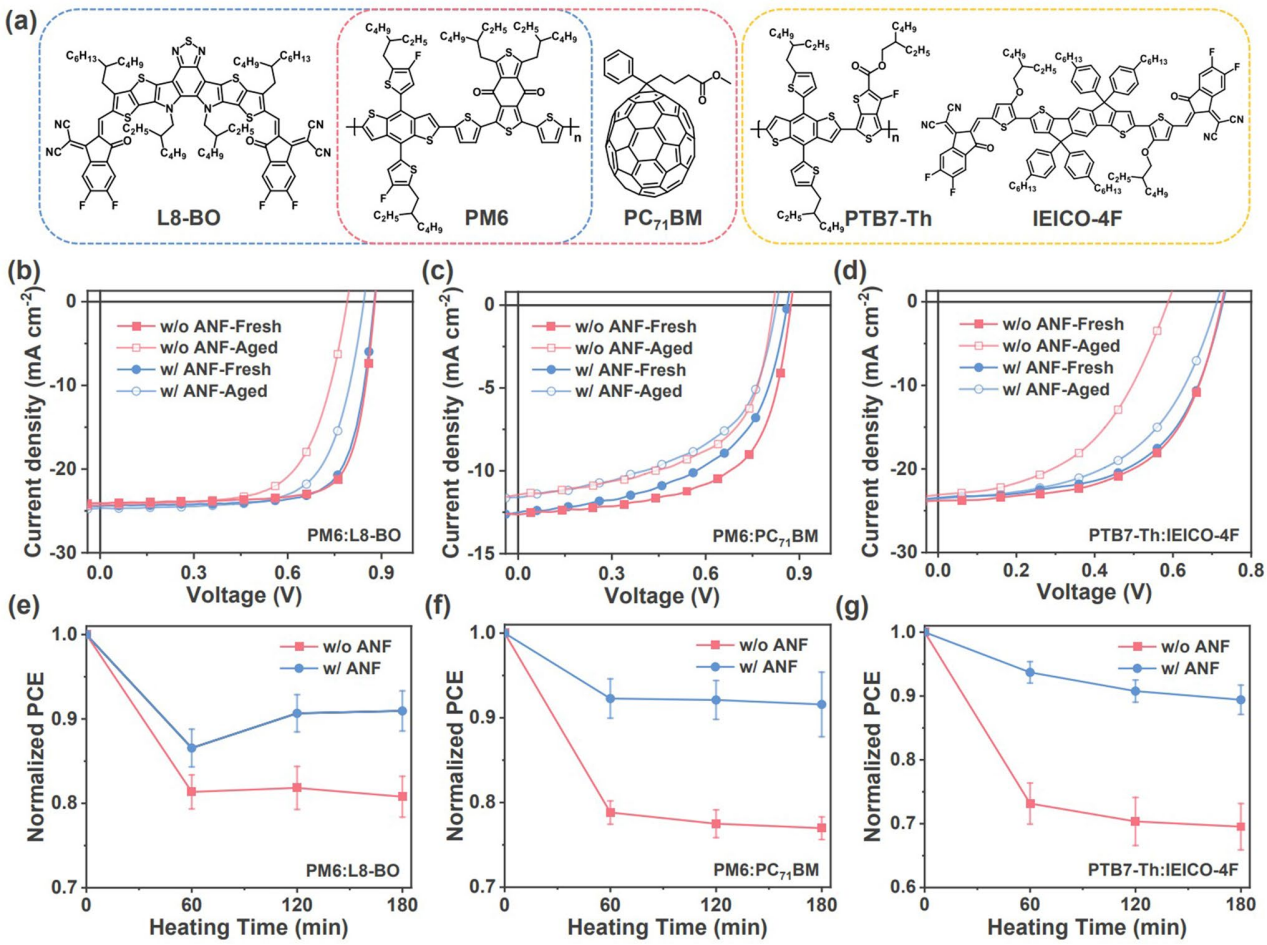
**a** Chemical structures of PM6, PTB7-Th, L8-BO, IEICO-4F, and PC_71_BM. *J*-*V* curves and thermal stability curves of devices without and with ANF under 130 °C in N_2_ atmosphere based on **b, e** PM6:L8-BO, **c, f** PM6:PC_71_BM, and **d, g** PTB7-Th:IEICO-4

To demonstrate the universality of this strategy, we extended our study to three other PV systems apart from PM6:BTP-eC9 (Fig. 6). The comprehensive photovoltaic parameters of the corresponding inverted devices are provided in Table S2. After heating to 130 °C are for 3 h, the PCE of ANF-free PM6:L8-BO devices decreased from 16.5% to 12.8%, and the PCE of ANF-free PM6:PC_71_BM devices decreased from 6.93% to 5.46%. In addition, the PCE of the ANF-free PTB7-Th:IEICO-4F devices decreased from 10.3% to 6.71%. These ANF-free devices retained only 80.78%, 76.96%, and 69.52% of their original PCE value, respectively. The incorporation of ANF networks into these non-fullerene and fullerene-based devices resulted in a significant improvement in thermal stability. After heating to 130 °C for 3 h, the PCE of the ANF-incorporated PM6:L8-BO device decreased from 16.2% to 14.6%, the PCE of the ANF-incorporated PM6:PC_71_BM device decreased from 6.01% to 5.15%, and the PCE of the ANF-incorporated PTB7-Th:IEICO-4F device decreased from 10.1% to 9.01%. Thus, these ANF-incorporated devices retained 90.95%, 89.91%, and 89.42% of their original average PCE value, respectively. Furthermore, the efficiency of these devices bearing ANF networks is comparable to that of ANF-free devices. These findings confirm the generality of the strategy of using rigid networks to stabilize the active layer morphology.

## Conclusions

In summary, we have developed and comprehensively investigated a universal strategy for enhancing the thermal stability of bulk heterojunction OPVs through the introduction of a polymer fiber rigid network with high *T*_g_ within the photoactive layer. ANF was chosen as a network block in this work. By optimizing the distribution of the ANF rigid network in the photoactive layer, we achieved a PCE of 16.9% in inverted BHJ OPVs based on PM6:BTP-eC9, comparable to that of the ANF-free counterpart. More importantly, the optimized ANF-incorporated OPVs exhibited excellent thermal stability when kept at 130 °C for 3 h, an important enhancement of the thermal stability of state-of-the-art organic photovoltaic systems. The concurrent high performance and thermal stability originate from the suppression of molecular diffusion and crystallization and the retention of the optimum phase separation in the BHJ films. Further characterization and analysis of the charge carrier dynamics showed that the frozen BHJ morphology effectively impeded the deterioration of exciton quenching, charge transport, and charge extraction properties during thermal aging, thereby improving the thermal stability of the devices bearing ANF networks. Besides, the beneficial role of the ANF rigid network was also confirmed for three other common organic photovoltaic systems, demonstrating that the strategy is universal. The proposed strategy offers valuable and unique perspectives for stabilizing the morphology of photoactive layers under thermal aging. Delving into alternative heat-resistant materials as component of the rigid network and optimizing the property of the rigid network can unleash the full potential of this strategy, fostering not only enhanced thermal stability, but also further improving mechanical resilience.

## Supplementary Information

Below is the link to the electronic supplementary material.

Supplementary file1 (DOCX 1860 KB)
