# Supplementary material for: Polymer Fiber Rigid Network with High Glass Transition Temperature Reinforces Stability of Organic Photovoltaics
Source: Nanomicro Lett. 2024 Jun 18;16:224. doi: 10.1007/s40820-024-01442-0 (PMC11189398; doi:10.1007/s40820-024-01442-0)
Supplement: Supplementary file 1 — Supplementary file1 (DOCX 1860 KB) [file 40820_2024_1442_MOESM1_ESM.docx]

Supporting Information for

**Polymer Fiber Rigid Network with High Glass Transition Temperature Reinforces Stability of Organic Photovoltaics**

Qiao Zhou^1^, Cenqi Yan^1,^ *, Hongxiang Li^1^, Zhendong Zhu^1^, Yujie Gao^1^, Jie Xiong^1^, Hua Tang^2^, Can Zhu^3^, Hailin Yu^1^, Sandra P Gonzalez Lopez^2^, Jiayu Wang^1^, Meng Qin^1^, Jianshu Li^1^, Longbo Luo^1,^ *, Xiangyang Liu^1^, Jiaqiang Qin^1^, Shirong Lu^4^, Lei Meng^3^, Frédéric Laquai^2^, Yongfang Li^3^, Pei Cheng^1,^ *

^1^ College of Polymer Science and Engineering, State Key Laboratory of Polymer Materials Engineering, Sichuan University, Chengdu, 610065, P. R. China

^2^ KAUST Solar Center, Physical Sciences and Engineering Division, King Abdullah University of Science and Technology (KAUST), Thuwal, Kingdom of Saudi Arabia

^3^ Beijing National Laboratory for Molecular Sciences, CAS Key Laboratory of Organic Solids, Institute of Chemistry, Chinese Academy of Sciences, Beijing, 100190, P. R. China

^4^ Department of Material Science and Technology, Taizhou University, Taizhou, 318000, P. R. China

*Corresponding authors. E-mail: [yancenqi@scu.edu.cn](mailto:yancenqi@scu.edu.cn) (Cenqi Yan); [luolongbo@scu.edu.cn](mailto:luolongbo@scu.edu.cn) (Longbo Luo); [chengpei@scu.edu.cn](mailto:chengpei@scu.edu.cn) (Pei Cheng)

Supplementary Figures and Tables


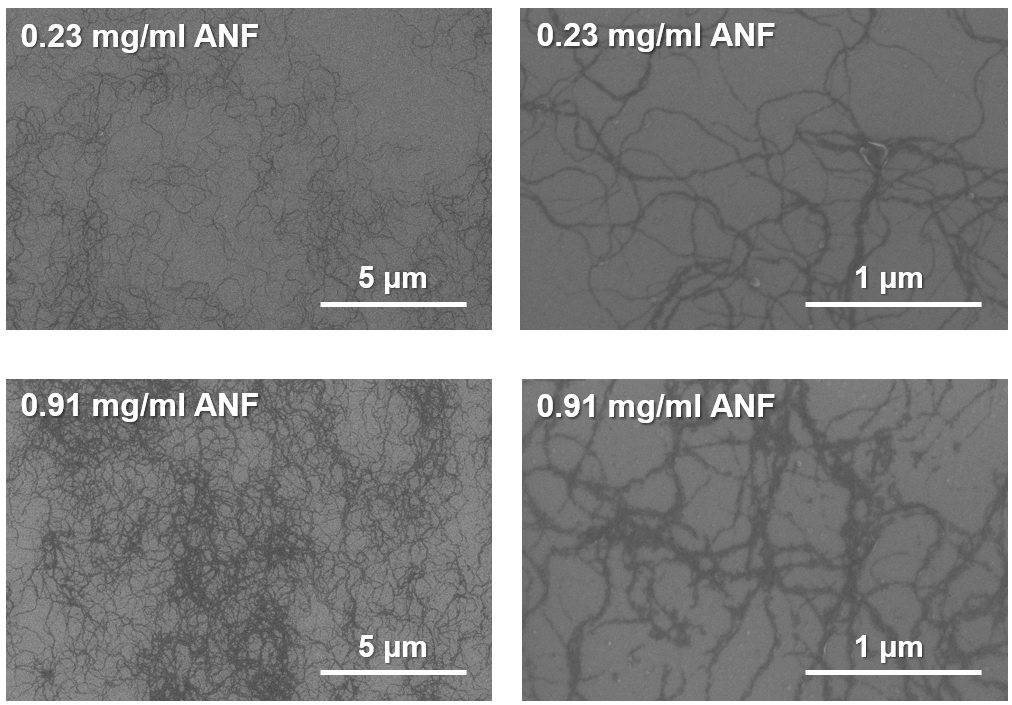


**Fig. S1** SEM images of ANF films with different dispersion concentrations


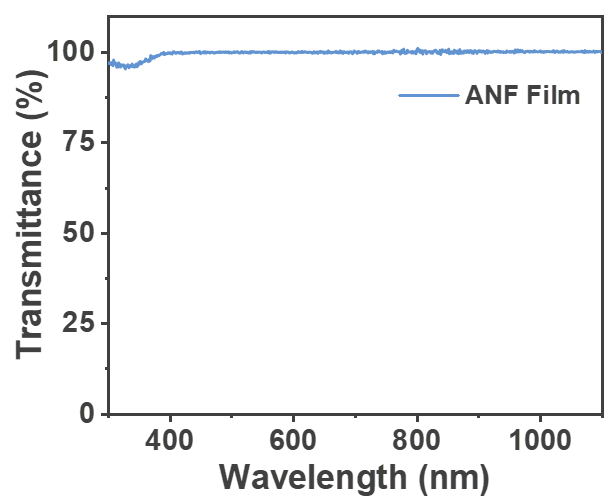


**Fig. S2** Transmittance of ANF film


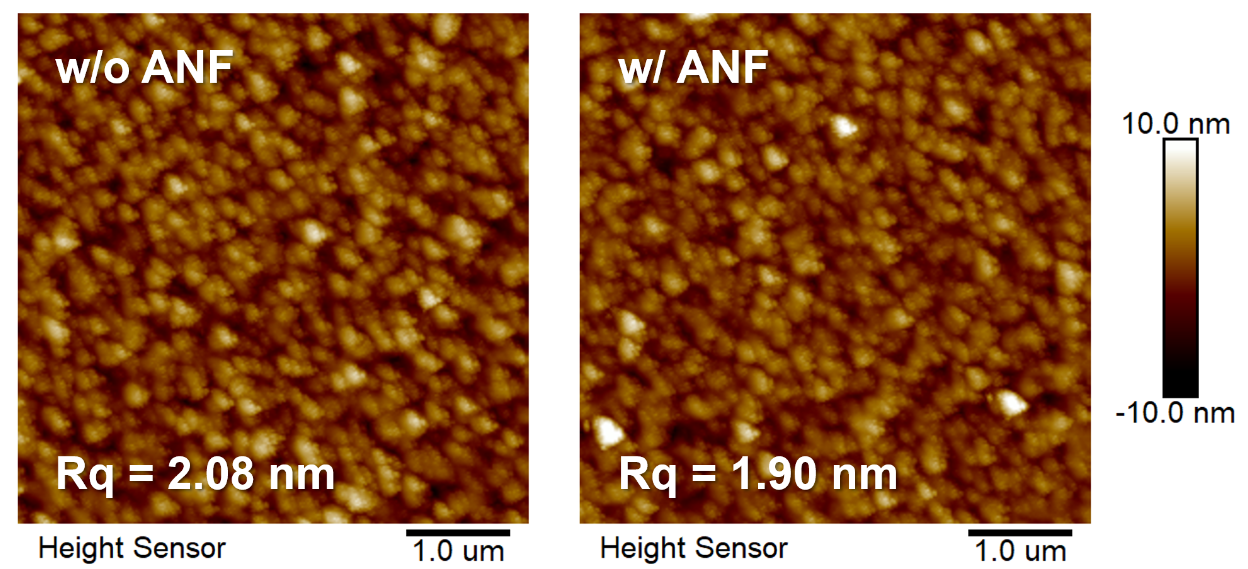


**Fig. S3** AFM images of active layers with and without ANF


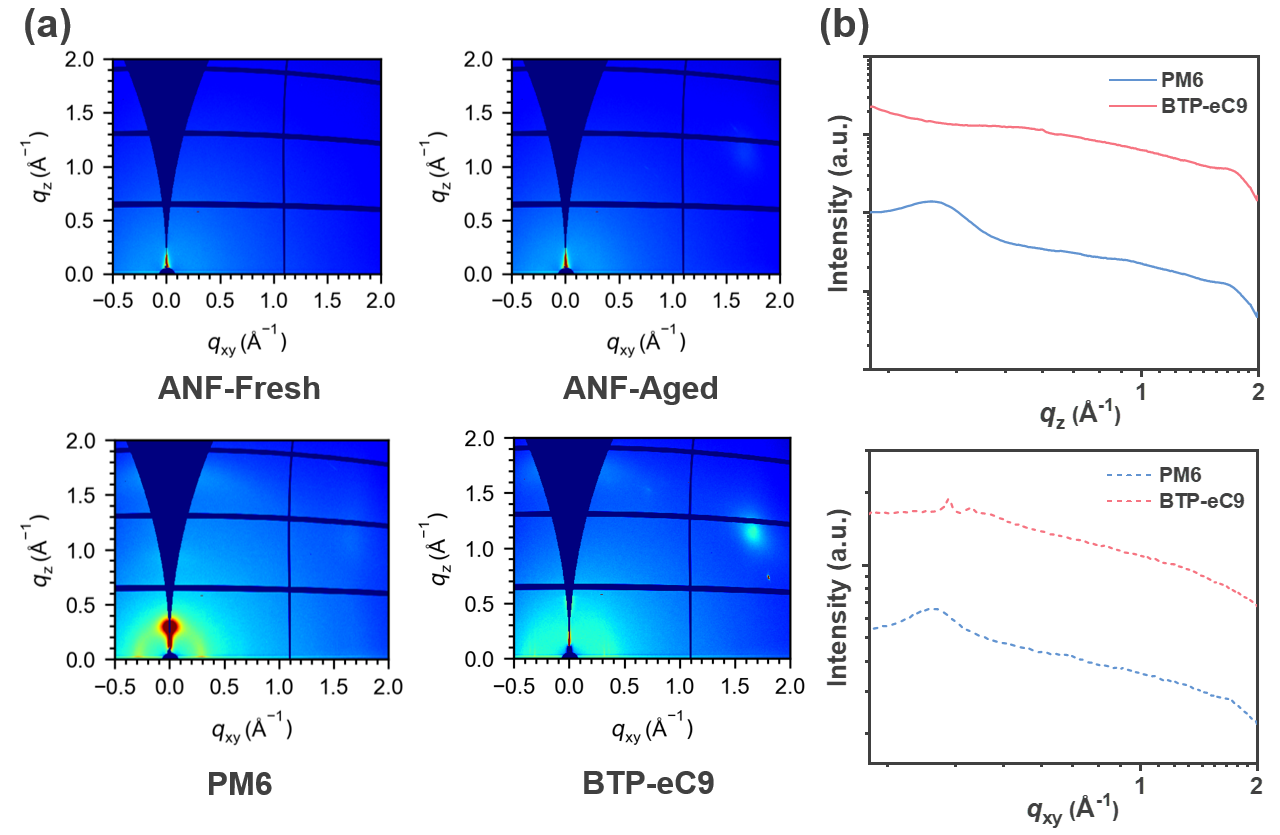


**Fig. S4 a** 2D and **b** 1D GIWAXS diffraction patterns of neat PM6, BTP-eC9, ANF-fresh and ANF-aged films


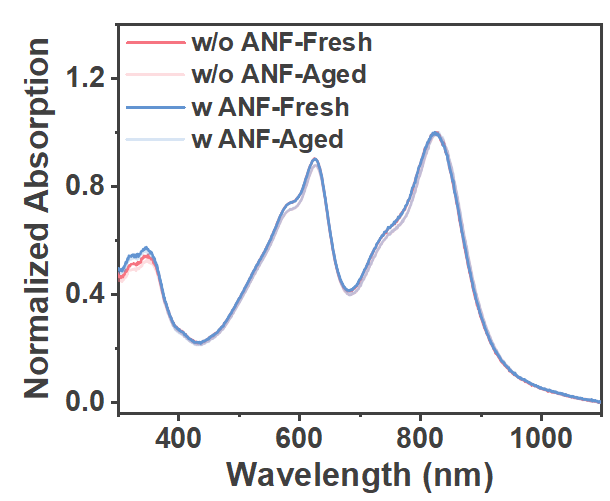


**Fig. S5** Normalized absorption spectra of PM6:BTP-eC9 blend films without and with ANF before and after heating at 130 °C for 3 h

**Table S1** Photovoltaic parameters decay of PM6: BTP-eC9-based OPVs with ANF of different dispersion concentrations heated at 130 °C for 0, 10, 20, 30, 60, 120, and 180 min

| ANF dispersion concentration (mg mL^-1^) | heating time (min) | remaining *V*_OC_ ratio ^a)^ | remaining  *J*_SC_ ratio ^a)^ | remaining  FF ratio ^a)^ | remaining  PCE ratio ^a)^ |
| --- | --- | --- | --- | --- | --- |
| 0 | 0 | 1.00 | 1.00 | 1.00 | 1.00 |
| 0 | 10 | 0.957 | 1.01 | 0.945 | 0.907 |
| 0 | 20 | 0.943 | 1.02 | 0.941 | 0.899 |
| 0 | 30 | 0.940 | 1.02 | 0.939 | 0.897 |
| 0 | 60 | 0.921 | 1.02 | 0.923 | 0.865 |
| 0 | 120 | 0.903 | 1.02 | 0.912 | 0.837 |
| 0 | 180 | 0.894 | 1.01 | 0.909 | 0.820 |
| 0.23 | 0 | 1.00 | 1.00 | 1.00 | 1.00 |
| 0.23 | 10 | 0.985 | 1.01 | 0.963 | 0.954 |
| 0.23 | 20 | 0.981 | 1.02 | 0.975 | 0.971 |
| 0.23 | 30 | 0.978 | 1.02 | 0.972 | 0.970 |
| 0.23 | 60 | 0.974 | 1.03 | 0.971 | 0.971 |
| 0.23 | 120 | 0.965 | 1.03 | 0.964 | 0.951 |
| 0.23 | 180 | 0.962 | 1.02 | 0.959 | 0.938 |
| 0.46 | 0 | 1.00 | 1.00 | 1.00 | 1.00 |
| 0.46 | 10 | 0.987 | 1.00 | 0.968 | 0.955 |
| 0.46 | 20 | 0.982 | 1.01 | 0.975 | 0.965 |
| 0.46 | 30 | 0.979 | 1.01 | 0.977 | 0.966 |
| 0.46 | 60 | 0.975 | 1.02 | 0.974 | 0.960 |
| 0.46 | 120 | 0.970 | 1.01 | 0.970 | 0.946 |
| 0.46 | 180 | 0.967 | 1.00 | 0.964 | 0.933 |
| 0.91 | 0 | 1.00 | 1.00 | 1.00 | 1.00 |
| 0.91 | 10 | 0.989 | 1.00 | 0.970 | 0.959 |
| 0.91 | 20 | 0.982 | 1.01 | 0.972 | 0.960 |
| 0.91 | 30 | 0.979 | 1.01 | 0.973 | 0.960 |
| 0.91 | 60 | 0.976 | 1.01 | 0.969 | 0.951 |
| 0.91 | 120 | 0.972 | 1.01 | 0.968 | 0.945 |
| 0.91 | 180 | 0.970 | 1.00 | 0.962 | 0.931 |

^a)^ Average value of eight independent devices

**Table S2** Average and best device data based on PM6: L8-BO, PM6: PC_71_BM, and PTB7-Th: IEICO-4F blend films without and with ANF before and after heating at 130 °C for 180 min

| active layer | ANF | heating time (min) | *V*_OC_ (V) | *J*_SC_  (mA cm^-2^) | FF (%) | PCE (%) | |
| --- | --- | --- | --- | --- | --- | --- | --- |
|  |  |  |  |  |  | best | average ^a)^ |
| PM6:L8-BO | w/o | 0 | 0.878 | 24.6 | 76.4 | 16.5 | 16.1 |
|  | w/o | 180 | 0.790 | 24.8 | 65.3 | 12.8 | 13.0 |
|  | w | 0 | 0.875 | 24.9 | 74.4 | 16.2 | 16.0 |
|  | w | 180 | 0.843 | 25.2 | 68.9 | 14.6 | 14.5 |
| PM6:PC_71_BM | w/o | 0 | 0.872 | 12.9 | 61.8 | 6.93 | 6.55 |
|  | w/o | 180 | 0.816 | 11.7 | 57.3 | 5.46 | 5.05 |
|  | w | 0 | 0.861 | 12.8 | 54.6 | 6.01 | 5.55 |
|  | w | 180 | 0.826 | 11.8 | 52.7 | 5.15 | 5.03 |
| PTB7-Th:IEICO-4F | w/o | 0 | 0.725 | 24.3 | 58.8 | 10.3 | 9.87 |
|  | w/o | 180 | 0.588 | 23.6 | 48.4 | 6.71 | 6.87 |
|  | w | 0 | 0.727 | 23.9 | 57.8 | 10.1 | 9.65 |
|  | w | 180 | 0.712 | 24.0 | 52.6 | 9.01 | 8.65 |

^a)^ Average value of eight independent devices
